# Droxidopa for symptomatic neurogenic orthostatic hypotension: what can we learn?

**DOI:** 10.1007/s10286-016-0394-2

**Published:** 2017-06-16

**Authors:** Horacio Kaufmann

**Affiliations:** Department of Neurology, Dysautonomia Center, New York University School of Medicine, 530 First Avenue, Suite 9Q, New York, NY 10017 USA

On February 18th 2014, the US Food and Drug Administration (FDA) approved droxidopa (Northera^®^), an orally active synthetic precursor of norepinephrine, for the treatment of symptomatic neurogenic orthostatic hypotension (nOH). It was the first new drug approval for nOH in almost 20 years. Two years before, the FDA Cardiovascular and Renal Drugs Advisory Committee had voted 7 out of 13 in favor of approval, but the FDA requested more data. With the results of a new clinical trial, a second Committee meeting voted almost unanimously to approve droxidopa (16 in favor out of 17). Droxidopa was approved for the treatment of symptomatic nOH in patients with primary autonomic failure, a group of disorders that includes Parkinson disease (PD), pure autonomic failure (PAF), multiple system atrophy (MSA), dopamine beta-hydroxylase deficiency, and non-diabetic autonomic neuropathies. PD, MSA, and PAF are now classified as synucleinopathies, while *non*-*diabetic autonomic neuropathy* is a broad term that includes autoimmune, genetic, and other autonomic neuropathies.

Droxidopa is not a new compound. It was first synthesized in 1919 by German chemists [9] who thought it could be a catecholamine precursor. In the late 1940s, Blaschko and colleagues in England showed that droxidopa could be converted to norepinephrine, in vivo, and that this step required the action of the enzyme DOPA decarboxylase, a.k.a. aromatic amino acid decarboxylase [1, 3, 4]. In 1989 it was reported that in patients with familial amyloid polyneuropathy and symptomatic nOH treatment with 600 mg of droxidopa increased plasma norepinephrine levels and standing blood pressure [10]. Subsequent studies in Japan led to its approval in 1989 for the treatment of nOH in patients with PD, MSA, and familial amyloid polyneuropathy [6].

**Fig. 1:**
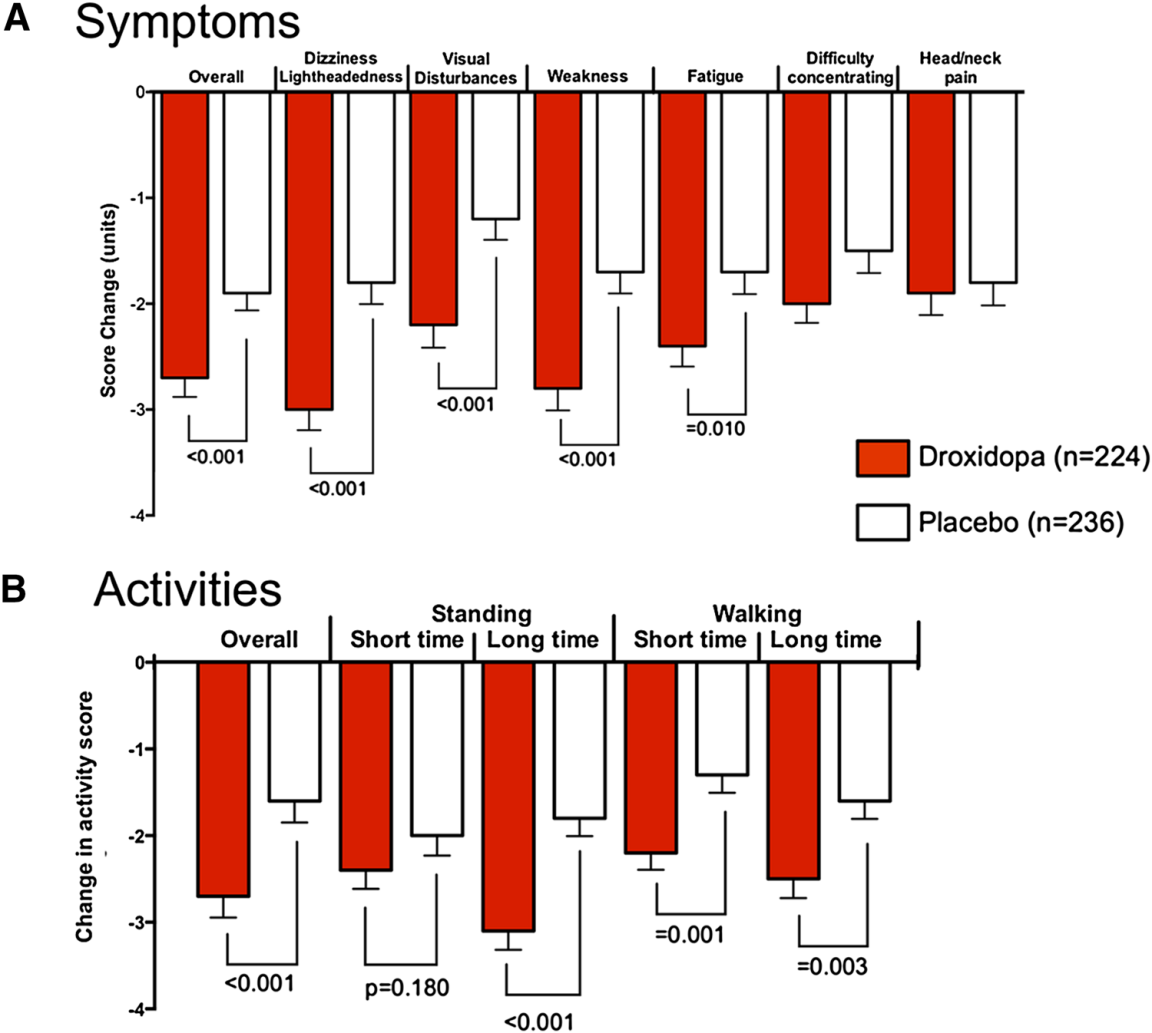
Mean score change from baseline to week 1 in the Orthostatic Hypotension Questionnaire (OHQ) from the integrated analysis of clinical trials of droxidopa. **a** Orthostatic Hypotension Symptoms Assessment (OHSA) and **b** Orthostatic Hypotension Daily Activity Scale (OHDAS). Score change on a rating scale from 0 (none/no interference) to 10 (worst possible/complete interference). A negative change represents a decrease in symptom burden

Eventually, three pivotal double blind clinical trials led to the FDA approval of droxidopa (Northera^®^) in the USA. The trials showed that patients with symptomatic nOH receiving droxidopa had both symptomatic improvement and higher blood pressure when standing than those on placebo. Each of these trials and the integrated analysis, in which almost 1000 patients were screened, have recently been published [2, 5, 8]. In summary, 226 patients received droxidopa and 236 received placebo. Symptoms of nOH were measured with a validated scale, the Orthostatic Hypotension Questionnaire [7], which assesses the presence of clinical manifestations of hypotension-related organ hypoperfusion including dizziness, lightheadedness, fatigue, or “coat-hanger” pain, on a scale from 0 (no symptoms/no interference) to 10 (worst possible/complete interference). The scale also includes measures of activity of daily living (i.e., how much interference the patient has when performing activities that require standing for a short time or for a long time). Those who received droxidopa improved in virtually all nOH symptom scores compared to those receiving placebo (Fig. 1). Droxidopa also increased upright systolic blood pressure significantly (+11.5 ± 20.5 mmHg vs. placebo +4.8 ± 21.0 mmHg; *p* < 0.001).

Clinical trials in a rare and clinically heterogeneous disorder like nOH pose a number of challenges. Recruitment can take a long time and the number of patients in each diagnostic category is always relatively small. With this limitation in mind, post hoc combined analysis of these trials showed a number of interesting leads. Droxidopa was particularly effective in patients with PD and PAF, but appeared less so in patients with MSA. The most puzzling finding is that among patients with MSA, treatment with droxidopa resulted in a larger decrease in symptom burden than in patients with other synucleinopathies; however, this improvement was not statistically better than placebo [2]. This is, perhaps, due to a greater placebo effect in this patient group, or to the presence of the Hawthorne effect, i.e., patients are particularly compliant with non-pharmacological measures to treat nOH (liberalization of salt and water, avoiding carbohydrates and alcohol, etc.) because they are participating in a clinical trial. It may also be related to the different site of pathology in MSA (central sympathetic denervation), in contrast to PD and PAF (mostly peripheral sympathetic denervation).

Another important issue is whether patients taking DOPA decarboxylase inhibitors (DDCI, e.g., carbidopa), which are always combined with levodopa in the treatment of PD, get less benefit from droxidopa. DDCI block the conversion of droxidopa to norepinephrine and could, theoretically, block droxidopa’s blood pressure-raising effect. In the integrated analysis, the magnitude of improvement observed in patients on droxidopa not taking DDCI was more pronounced than in those taking DDCI [10]. However, because none of the studies were designed to specifically assess this, no statistical model could confirm the significance of the difference. Moreover, the dosages of DDCI were not collected and the dose–response effect could not be analyzed. The combined analysis did show, however, that patients receiving droxidopa still had an increase in their blood pressure and symptomatic improvement when taking DDCI at clinically indicated dosages.

During the trials, droxidopa was given in a fixed three times a day schedule. Now that the drug has been on the market for some time, clinical experience suggests that a better strategy is to use different dosages of droxidopa throughout the day. A higher dosage in the morning when blood pressure standing is at its lowest is a strategy used by most experienced clinicians when treating symptomatic nOH.

Droxidopa has been available in the USA for almost 4 years now. For many patients with different autonomic disorders droxidopa is well tolerated and improves their symptoms of nOH and quality of life. Still, like with any drug, some patients fail to respond. Why droxidopa appears not to be effective in this subpopulation remains an important clinical and research question. It is also important to remember that, in order to be effective, droxidopa must be given with clear explanations of the need for non-pharmacologic measures, which constitute the necessary background for all pressor agents to exert their beneficial effect in patients with symptomatic nOH.

In this special supplement of *Clinical Autonomic Research* entitled “Neurogenic orthostatic hypotension: grand rounds”, eight practicing clinicians from different medical centers across the USA discuss their own real-life experience in treating patients with nOH, when and how to start droxidopa, and several challenging situations. This supplement also includes a basic glossary with information on treatments of nOH and supine hypertension, based on a recent expert consensus criteria and other publications.

Our hope is that this basic information is useful to our readership and can assist them in managing their patients with nOH.
